# A Time Series Analysis of Air Pollution and Preterm Birth in Pennsylvania, 1997–2001

**DOI:** 10.1289/ehp.7646

**Published:** 2005-02-02

**Authors:** Sharon K. Sagiv, Pauline Mendola, Dana Loomis, Amy H. Herring, Lucas M. Neas, David A. Savitz, Charles Poole

**Affiliations:** ^1^Department of Epidemiology, University of North Carolina at Chapel Hill, Chapel Hill, North Carolina, USA;; ^2^National Health and Environmental Effects Research Laboratory, Office of Research and Development, U.S. Environmental Protection Agency, Research Triangle Park, North Carolina, USA;; ^3^Department of Biostatistics, University of North Carolina at Chapel Hill, Chapel Hill, North Carolina, USA

**Keywords:** air pollution, environmental epidemiology, particulate matter, pregnancy, preterm birth, sulfur dioxide

## Abstract

Preterm delivery can lead to serious infant health outcomes, including death and lifelong disability. Small increases in preterm delivery risk in relation to spatial gradients of air pollution have been reported, but previous studies may have controlled inadequately for individual factors. Using a time-series analysis, which eliminates potential confounding by individual risk factors that do not change over short periods of time, we investigated the effect of ambient outdoor particulate matter with diameter ≤10 μm (PM_10_) and sulfur dioxide on risk for preterm delivery. Daily counts of preterm births were obtained from birth records in four Pennsylvania counties from 1997 through 2001. We observed increased risk for preterm delivery with exposure to average PM_10_ and SO_2_ in the 6 weeks before birth [respectively, relative risk (RR) = 1.07; 95% confidence interval (CI), 0.98–1.18 per 50 μg/m^3^ increase; RR = 1.15; 95% CI, 1.00–1. 32 per 15 ppb increase], adjusting for long-term preterm delivery trends, co-pollutants, and offsetting by the number of gestations at risk. We also examined lags up to 7 days before the birth and found an acute effect of exposure to PM_10_ 2 days and 5 days before birth (respectively, RR = 1.10; 95% CI, 1.00–1.21; RR = 1.07; 95% CI, 0.98–1.18) and SO_2_ 3 days before birth (RR = 1.07; 95% CI, 0.99–1.15), adjusting for covariates, including temperature, dew point temperature, and day of the week. The results from this time-series analysis, which provides evidence of an increase in preterm birth risk with exposure to PM_10_ and SO_2_, are consistent with prior investigations of spatial contrasts.

Preterm delivery can lead to neonatal mortality as well as an array of infant morbidities that range from pulmonary to neurologic outcomes (Martin et al. 2002). The prevalence of preterm delivery was 11.6% in the United States in the year 2000 (Centers for Disease Control and Prevention 1999; Martin et al. 2002). Fewer than half of preterm births in developed countries can be attributed to known risk factors (Berkowitz and Pepiernik 1993; Kramer 1987). There is a clear need to explore causes of preterm delivery that might be modifiable, such as environmental exposures.

A small number of previous studies have explored the association between criteria air pollutants—carbon monoxide, nitrogen dioxide, ozone, sulfur dioxide, particulate matter with diameter ≤10 μm (PM_10_), and in some cases total suspended particulates (TSP)—and preterm delivery by examining spatial exposure contrasts between individuals based on the place of residence at birth (Bobak 2000; Liu et al. 2003; Maroziene and Grazuleviciene 2002; Ritz et al. 2000; Xu et al. 1995). Although these studies varied with regard to the pollutant(s) evaluated, the most consistent findings reported were for positive associations between PM_10_/TSP and SO_2_ late in pregnancy and preterm delivery (Bobak 2000; Liu et al. 2003; Ritz et al. 2000; Xu et al. 1995).

Relying on spatial analyses comparing high-exposure areas with low-exposure areas, previous studies have been subject to the effects of confounding by individual risk factors, such as maternal smoking and unmeasured or unknown risk factors. To date, no study has looked at the effect of air pollution on preterm delivery using a time-series analysis with exposure contrasts over time rather than space. Time-series analysis removes the influence of covariates that vary across individuals but not within individuals over short periods of time.

We investigated the associations during the years 1997 through 2001 of risk for preterm delivery with average concentrations of ambient outdoor PM_10_ and SO_2_ during the 6 weeks preceding birth in four Pennsylvania counties selected for their contrasting PM_10_ and SO_2_ distributions. We also explored a possible acute effect of these pollutants by narrowing the exposure window to a 1-day interval before birth, with daily lags ranging from 1 to 7 days.

## Materials and Methods

### Study population.

The study population consisted of all live singleton births whose mothers resided in four Pennsylvania counties (Allegheny, Beaver, Lackawanna, and Philadelphia) and for whom birth certificates were filed with dates of birth between 1 January 1997 and 31 December 2001 (*n* = 187,997). These counties were chosen for their diverse mix and wide range of pollutants. Births were restricted to gestational ages within the range of 20–44 weeks because births at < 20 gestational weeks are rarely viable and births are usually induced before 44 weeks of gestation.

### Exposure estimation.

We obtained ambient outdoor air pollution monitoring data for the four study counties over the 5-year study period (20 November 1997 through 30 December 2001) from the U.S. Environmental Protection Agency (EPA) Air Quality System (AQS). Daily mean values were computed for the two air pollutants of primary interest (PM_10_ and SO_2_) and for those considered co-pollutants (CO, NO_2_, and O_3_). PM_10_ was collected hourly for three of the counties using a tapered element oscillating microbalance sampler, and every sixth day in one county using a high-volume air sampler system. We used public access data from EPA’s AQS and did not directly measure pollutants with these samplers. Thus we do not know the manufacturer for the TEOM and high-volume air samplers. Three counties had multiple monitoring stations for each pollutant; a single average countywide daily pollutant value was computed for each of these counties.

Because the acute effects analysis assessed shorter-term changes in pollution levels, we considered covariates such as temperature and dew point temperature (a measure of relative humidity), which also change over short periods of time. These meteorologic data were obtained from the National Weather Service (NOAA/NCDC 2003).

### Outcome assessment.

Gestational age was computed as the number of weeks between the date of the last menstrual period (LMP) and the date of birth. For birth records missing the date of the LMP, the clinical estimate of gestation was used. Eligible births with gestational ages < 36 weeks were considered preterm. Counts of preterm births were tallied for each day during the 5-year observation period.

### Statistical analysis.

We conducted a Poisson regression analysis, which followed PM_10_ and SO_2_ levels and counts of preterm births jointly over time. Preterm births were measured as a daily count of events assumed to be independent and random. The Poisson model was selected because these counts were small on any given day. Because days of observation were the units of analysis, exposure gradients were measured with reference to time rather than to other individuals.

Covariates in the final models, including co-pollutants, long-term trends in preterm birth, and weather (temperature and dew point temperature), were included *a priori* because of their established temporal relationship with air pollution in the previous literature and their likely associations with the air pollutants of interest. Previous studies also suggest a consistent seasonal pattern for preterm birth (Cooperstock and Wolfe 1986; Keller and Nugent 1983; Matsuda and Kahyo 1992).

#### Mean 6-week analysis.

We used daily pollutant levels in each county to compute the mean air pollution concentration value for the 6-week period preceding each day of observation. To incorporate county-level information, we used a multivariable mixed-effects model with a random intercept for each county. We controlled for long-term preterm birth trends and mean 6-week level of co-pollutants (CO, NO_2_, and SO_2_ in the PM_10_ analysis and CO, NO_2_, and PM_10_ in the SO_2_ analysis). Because the number of births in the population varies by season, we included a term in the time-series model to offset the total number of gestations in the population at risk for preterm birth on each day during the study period.

We examined county-specific long-term trends for preterm birth using LOESS plots, which are nonparametric, locally weighted regression smoothers. Parametric functions were fit in the final models, however, to achieve more conservative standard errors yet account for long-term trends in a flexible manner (Dominici et al. 2002). To control for these trends in the multivariable model, we fit spline functions with knots placed at points that parsimoniously simulated the LOESS smoothing curves. Various spline functions, including linear, quadratic, and cubic splines, were evaluated, and the function that provided the best fit was retained.

#### Daily acute effect analysis.

For the analysis of the acute effect of PM_10_ and SO_2_, we investigated risk for preterm birth in relation to levels of pollutants for a 1-day exposure window with lags from 1 to 7 days before birth. In addition to adjusting for long-term trends, we controlled for daily levels of co-pollutants and other short-term time-varying covariates, including temperature, dew point temperature, and day of the week. The number of gestations at risk for preterm birth was used as an offset. We explored different exposure windows and lags for both temperature and dew point temperature; because associations between these covariates and preterm birth have not been reported in the literature, we chose the window and lag combination with the best fit, determined by Akaike’s Information Criterion (Akaike 1973). Linear relationships of temperature and dew point temperature with preterm birth were improbable; we therefore used parametric spline functions to represent these covariates.

We estimated relative risks (RRs) and 95% confidence intervals (CIs) for 6-week and daily acute exposure (for each of the 7 days before birth) to PM_10_ and SO_2_. Pollutants were modeled as continuous variables, and estimates were reported for exposure increments of 50 μg/m^3^ in PM_10_ and 15 ppb in SO_2_. These ranges were chosen because they were consistent with previous studies yet still within the range of exposure for our study; this facilitated the direct comparison of our results with the existing literature. The possibility of a nonlinear dose–response relationship between pollutants and preterm birth was also assessed by categorically dividing pollutant concentration into quarters. RRs and 95% CIs were estimated for each quarter.

Approval for this research was obtained from the Committee on the Protection of the Rights of Human Subjects at the Biomedical Institutional Review Board of the University of North Carolina at Chapel Hill’s School of Medicine.

## Results

### Descriptive statistics.

A total of 200,253 birth records were available for the four counties between 1997 and 2001. For 36,839 birth records missing the date of the LMP, we used the clinical estimate of length of gestation; secondary analyses showed that inclusion of these births did not meaningfully change the estimated associations but did enhance precision, and we therefore included these births in the final analyses. We excluded 677 births to mothers whose geocoded residence at delivery was outside the four study counties and 6,322 multiple births. An additional 5,257 births with gestational ages outside the range of 20–44 weeks were excluded. After these exclusions, 187,997 births (94% of the total) remained. Table 1 shows the demographic characteristics of this final study population. The exclusions did not substantially change the distribution of the demographic characteristics (results not shown).

### Mean 6-week exposure models.

Table 3 displays estimates of the association of mean 6-week PM_10_ and SO_2_ exposure and risk for preterm delivery. Risk for preterm birth increased with exposure to mean 6-week PM_10_ (RR = 1.07; 95% CI, 0.98–1.18 per 50-μg/m^3^ increase in PM_10_), offsetting by the total number of gestations at risk, and adjusting for county-specific long-term trends and co-pollutants (CO, NO_2_, and SO_2_). The RRs for the two higher PM_10_ exposure quarters were slightly elevated, but there was no clear evidence of a strictly monotonic dose–response relationship between 6-week PM_10_ exposure and preterm delivery in the quartile analysis.

### Acute effects (daily) exposure models.

**Figure 1:**
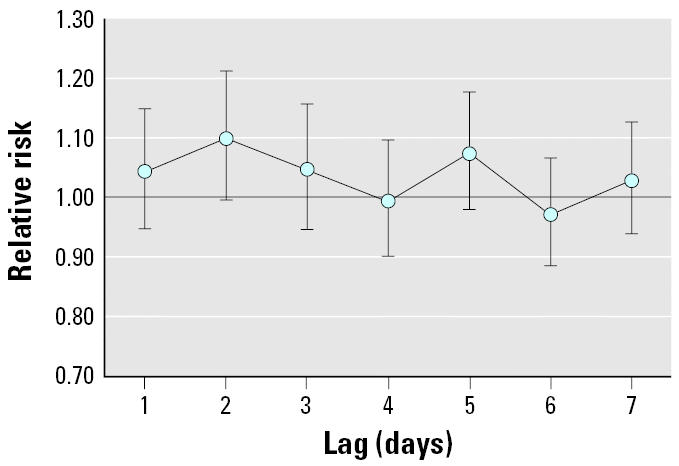
RRs and 95% CIs for preterm birth per 50-μg/m^3^ increase in PM_10_, lagged 1–7 days before birth, offsetting by the number of gestations at risk and adjusting for long-term trends, temperature, dew point temperature, day of the week, and co-pollutants (NO_2_, CO, and SO_2_) in four Pennsylvania counties, 1997–2001.

Among the 1-day acute time windows examined, preterm birth was most strongly associated with PM_10_ levels using a 2-day lag (adjusted RR = 1.10; 95% CI, 1.00–1.21 per 50 μg/m^3^) and a 5-day lag (adjusted RR = 1.07; 95% CI, 0.98–1.18 per 50 μg/m^3^) (Figure 1).

**Figure 2:**
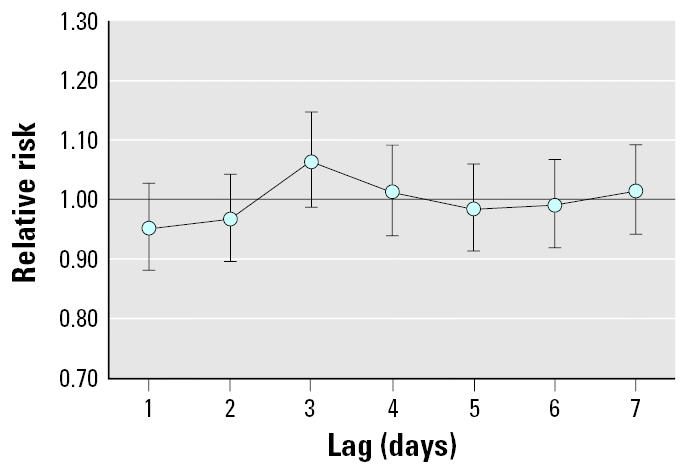
RRs and 95% CIs for preterm birth per 15-ppb increase in SO_2_, lagged 1–7 days before birth, offsetting by the number of live births and adjusting for long-term seasonal trend, temperature, dew point temperature, day of the week, and co-pollutants (NO_2_, CO, and PM_10_) in four Pennsylvania counties, 1997–2001.

For SO_2_, the lag with the strongest association was 3 days (RR = 1.07; 95% CI, 0.99–1.15 per 15 ppb) (Figure 2).

## Discussion

We observed an increased risk for preterm delivery during the last 6 weeks of pregnancy with exposure to SO_2_ and PM_10_. Our point estimates are similar to those reported in the literature, and the widths of our CIs compare favorably with those for previously reported estimates. The estimated number of excess preterm births that can be attributed to these pollutants, computed as the number needed to treat (NNT) (Cordell 1999), was approximately 1 excess preterm birth for every 125 births exposed to a 50 μg/m^3^ increase in PM_10_, and 1 excess preterm birth for every 58 births exposed to a 15 ppb increase in SO_2_. To compute the NNT, we assumed that the baseline prevalence approximated the overall prevalence of preterm birth in the study population [risk among unexposed (*R*_0_) = 0.114] and given a RR = 1.07 for PM_10_, the risk among exposed (*R*_1_) = 0.122, the RD (risk difference) = 0.008, and the NNT = 1/0.008 = 125. In the week before birth, the strongest associations were observed with a 2-day and 5-day lag for PM_10_ and a 3-day lag for SO_2_.

Several hypothesized mechanisms support biologic plausibility of an effect of air pollution on preterm birth. Two mechanisms potentially act through distinct pathways operating at the end of pregnancy. Changes in blood viscosity due to inflammation as a result of exposure to PM and SO_2_ have been observed (Peters et al. 1997). Inflammation has also been related to preterm delivery and could be associated with inadequate placental perfusion (Knotternus et al. 1990; Zondervan et al. 1987). This pathway could explain an acute effect of air pollution on preterm birth, evidence for which was observed for both PM_10_ and SO_2_.

A second possible pathway is a more long-term process that involves maternal infection during pregnancy. Although air pollution does not directly cause maternal infections, exposure to specific pollutants may impair immune function, which could enhance susceptibility to infection (Gardner 1984; Hertz-Picciotto et al. 2002). Subtle changes in the immune system could result in changes among vaginal flora, which promote vaginal pathogens associated with bacterial vaginosis, a risk factor for preterm birth (Minkoff et al. 1984). Studies also show associations between preterm labor and delivery and systemic maternal infections, such as pneumonia and pyelonephritis, as well as more local infections, such as intra-amniotic and urinary infections (Benedetti et al. 1982; Cunningham et al. 1973; Fan et al. 1987; Guzick and Winn 1985; Madinger et al. 1989; Moller et al. 1984; Naeye and Peters 1980; Pankuch et al. 1984; Regan et al. 1981; Romero and Mazor 1988). This pathway could explain the associations observed in this study for both PM_10_ and SO_2_ in the 6 weeks before birth.

Our results are consistent with a number of prior studies of PM_10_ and SO_2_ exposure and preterm birth, all of which examined spatial gradients in exposure. A retrospective cohort study of 97,158 infants born between 1989 and 1993 in Southern California found an increase in risk of preterm delivery with exposure to PM_10_ during the 6-week period preceding birth (RR = 1.20; 95% CI, 1.09–1.33 per 50-μg/m^3^ increase in PM_10_) (Ritz et al. 2000). This study did not investigate or control for the effect of SO_2_. A population-based prospective cohort study of 25,370 Chinese women that gave birth in Beijing in 1988 found evidence of an acute effect of TSP on preterm birth in the 7 days before birth [odds ratio (OR) = 1.10; 95% CI, 1.01–1.20] for each 100-μg/m^3^ increase in TSP (Xu et al. 1995). This study also reported evidence of an acute effect of SO_2_ in the 7 days before birth (OR = 1.21; 95% CI, 1.01–1.45) for each 100-μg/m^3^ (37.5 ppb) increase in SO_2_. A retrospective study conducted among 126,752 singleton births in the Czech Republic during 1991 reported adjusted ORs for preterm delivery to be 1.12 (95% CI, 0.97–1.28) and 1.24 (95% CI, 1.13–1.36) per 50-μg/m^3^ (18.75 ppb) increase in TSP and SO_2_, respectively, during the third trimester of pregnancy (Bobak 2000). In Vancouver, Canada, a retrospective study of all live births between 1985 and 1998 that did not investigate or control for particles found an association between preterm birth and SO_2_ (OR = 1.09; 95% CI, 1.01–1.19 per 5-ppb increase) during the last month of pregnancy (Liu et al. 2003).

A potential limitation of these previous studies is inadequate control for confounding by individual risk factors. Birth record data do not include information on all risk factors that could potentially confound the relationship between air pollution and preterm delivery. In addition, variables that are collected may be poorly measured or recorded, particularly when they come from a birth certificate, which could lead to residual confounding in either direction of the association between air pollution and preterm birth.

By observing the population over time using time-series analysis, we could investigate the impact of air pollutants without the influence of known and unknown individual risk factors that do not vary over short periods of time. Consistency of the overall findings of our study with previous studies suggests that confounding at the individual level is probably not explaining the observed association between air pollution and preterm delivery. However, more complete control of confounding could explain the weaker associations observed in this study; this could also be caused by differences in pollution levels or sources between study locations.

Our study was efficient in that it made use of existing air pollution and birth record data. This allowed for a very large, population-based study that is not subject to selection biases that arise when recruiting a patient population. In addition, the long study period and large population enhanced the precision of the estimated measures of effect. Given these advantages, in addition to the ease associated with using extant data sources relative to generating original data, investigation of additional health outcomes (including other birth outcomes) using this study design is advisable.

An acknowledged limitation of all studies of ambient air pollution is that measurements from stationary outdoor monitors may not represent individual exposure. Studies of air pollution and mortality, cardiovascular disease, and other respiratory outcomes have addressed this issue and report that, although relatively crude, ambient measures are often the most feasible measure of exposure in terms of cost and burden to the study participant (Samet et al. 2000). In addition, investigators project that the likely consequence of using ambient concentrations is to underestimate air pollution effects (Zeger et al. 2000).

The time-series analysis assumes that ambient pollution levels and personal exposure are often correlated over time, so although their absolute levels may be different, both will be high on a high-air-pollution day and low on a low-air-pollution day (Zeger et al. 2000). Measurement of outcomes in relation to time should therefore produce the appropriate exposure contrasts. Measurement error was probably nondifferential with respect to preterm delivery, which would most likely lead to underestimation of the true effect. Finally, studying ambient levels has advantages for informing regulations at the population level.

This study was limited to the investigation of two criteria pollutants collected by the U.S. EPA’s AQS (Air Quality System 2003). The question of whether PM_10_ and SO_2_ are hazardous themselves or are markers for other hazardous airborne pollutants, such as sulfates that result from SO_2_ emissions in the presence of water and oxygen, fine particles (PM_2.5_), or other noncriteria pollutants such as polycyclic aromatic hydrocarbons, was beyond the scope of this study and is a direction for further investigation. Some of the previous literature suggests associations between CO and/or NO_2_ and preterm birth (Bobak 2000; Liu et al. 2003; Maroziene and Grazuleviciene 2002; Ritz et al. 2000). We focused on PM_10_ and SO_2_ because the literature was the most consistent for these pollutants; however, we did perform preliminary analyses for CO, and NO_2_ and our results did not support an association between these pollutants and preterm birth.

Missing data for PM_10_ limited the number of observation days for both the 6-week and daily acute effects analysis, reducing the precision of the effect estimates.

We limited our analyses to exposure windows at the end of pregnancy. It is plausible, however, that air pollution could interfere with development of the placenta or other processes in early fetal development that begin a path to preterm delivery. Two previous studies found an association between exposure to PM_10_/TSP at beginning of pregnancy (first month and first trimester) and preterm delivery (Bobak 2000; Ritz et al. 2000). One of these studies also found an association between SO_2_ at the beginning of pregnancy and preterm delivery (Bobak 2000); however, these results were contradicted in another study (Liu et al. 2003).

We also did not adjust for other time windows of exposure in our analyses. For example, in the model of exposure to air pollution in the 6 weeks preceding birth, we did not control for exposure to air pollution earlier in pregnancy. In addition, we did not adjust for exposure during the 7 days preceding birth. This may have limited our ability to attribute effects to a specific time window of exposure, which would help to understand the biologic mechanism for a possible effect.

Gestational age, which is difficult to measure accurately, may have been incorrectly estimated on the birth certificate. We restricted our analysis to births between 20 and 44 weeks of gestation to attempt to filter out the most serious of these errors; however, it is likely that a degree of error remains. Error in gestational age is probably not influenced by air pollution, however, thus the misclassification is likely to be non-differential with respect to exposure, which most likely led to an attenuated estimate of the true effect of air pollution on preterm delivery.

Despite limitations in our data, we observed evidence of an effect of both PM_10_ and SO_2_ on preterm delivery. The absolute increases in risk were small, with 1 or 2 excess preterm births for every 100 births exposed to a 50-μg/m^3^ increase in PM_10_ or 15-ppb increase in SO_2_. However, many people live in urban centers and are chronically exposed to high levels of air pollution; if these small effects are indeed causal, the public health impact could be considerable. Additional studies in other geographical areas and time periods would be warranted. Further research to identify the critical time window(s) during pregnancy for the effect of air pollution on preterm birth, and to delineate the biologic mechanism for such an effect would also be useful.

## Figures and Tables

**Table 1:** Demographics of the study population*^a^* (*n* = 187,997) of births in four Pennsylvania counties, 1997–2001.

Variable	No. (%)
Preterm births (< 37 weeks)	21,450 (11.4)
Maternal age (years)	
< 15	690 (0.4)
15–19	24,737 (13.2)
20–24	43,096 (22.9)
25–29	48,515 (25.8)
30–34	44,823 (23.8)
35–39	21,777 (11.6)
≥40	4,325 (2.3)
Unknown	34 (0)
Maternal race	
White	111,084 (59.1)
African American	66,022 (35.1)
Asian	7,324 (3.9)
Other	1,096 (0.6)
Unknown	2,471 (1.3)
Maternal education (years)	
0–8	3,515 (1.9)
9–11	29,247 (15.6)
12	64,353 (34.2)
13–15	39,328 (20.9)
≥16	27,862 (14.8)
Unknown	23,692 (12.6)
Marital status	
Married to father	97,216 (51.7)
Not married to father	90,617 (48.2)
Unknown	164 (0.1)

a Includes all live singleton births with a nonmissing gestational age estimate on the birth certificate in the range of 20–44 weeks, excluding births with geocoded maternal address outside of the four study counties.

**Table 2:** Exposure statistics during the 5-year study period (1997–2001) for four counties in Pennsylvania.

Exposure	No. of observations	Range	Mean ± SD	Median
6-week PM_10_ (μg/m^3^)	5,851	8.7–68.9	27.1 ± 8.3	26.0
Daily PM_10_ (μg/m^3^)	4,204	2.0–156.3	25.3 ± 14.6	21.6
6-week SO_2_ (ppb)	7,304	0.8–17.0	7.9 ± 3.5	8.1
Daily SO_2_ (ppb)	7,296	0–54.1	7.9 ± 6.2	6.4

**Table 3:** RRs and 95% CIs for preterm birth and exposure to PM_10_ and SO_2_ in the 6 weeks preceding birth in four Pennsylvania counties, 1997–2001.

Pollutant	Range	RR*^a^* (95% CI)
PM_10_ (μg/m^3^)		
Continuous (per 50-μg/m^3^ increase)		1.07 (0.98–1.18)
First quarter	8.7–21.1	1.00
Second quarter	21.1–26.0	1.00 (0.95–1.05)
Third quarter	26.0–31.6	1.04 (0.99–1.09)
Fourth quarter	31.6–68.9	1.03 (0.98–1.08)
SO_2_ (ppb)		
Continuous (per 15-ppb increase)		1.15 (1.00–1.32)
First quarter	0.8–4.9	1.00
Second quarter	4.9–8.1	1.02 (0.97–1.06)
Third quarter	8.1–10.6	1.04 (0.98–1.10)
Fourth quarter	10.6–17.0	1.06 (0.99–1.14)

a RR offsetting by gestations at risk and adjusting for long-term seasonal preterm birth trend and co-pollutants (NO_2_, CO, and SO_2_ in the PM_10_ analysis, PM_10_ in the SO_2_ analysis).
